# Nesting, Sex Ratio and Natural Enemies of the Giant Resin Bee in Relation to Native Species in Europe

**DOI:** 10.3390/insects12060545

**Published:** 2021-06-11

**Authors:** Sara Straffon Díaz, Luca Carisio, Aulo Manino, Paolo Biella, Marco Porporato

**Affiliations:** 1 Department of Agricultural Forest and Food Sciences, University of Turin, Largo Paolo Braccini 2, 10095 Grugliasco (Turin), Italy; luca.carisio@unito.it (L.C.); aulo.manino@unito.it (A.M.); marco.porporato@unito.it (M.P.); 2 Department of Biotechnology and Biosciences, University of Milano-Bicocca, Piazza della Scienza 2, 20126 Milano, Italy; paolo.biella@unimib.it

**Keywords:** exotic bee, wild bees, *Megachile sculpturalis*, bee invasion, nesting behavior, trap nest, competition, sex ratio, natural enemies

## Abstract

**Simple Summary:**

Alien bee species could have detrimental effects; in particular, they may compete with native bee species for floral resources or nesting sites. Here, we first studied the interaction in artificial trap nests, installed in a semi-urban area of north-western Italy, between the first exotic bee in Europe, *Megachile sculpturalis*, and native wild bees. Second, we evaluated the performance of the exotic bee by means of the sex ratio, and we screened for the presence of natural enemies affecting its brood. Our results showed that *M. sculpturalis* brood cells cohabited tunnels with the native *Osmia cornuta.* Given the exotic cells’ position within tunnels and their resin-based material, exotic cells may act as a block for native bee emergence. Moreover, our study revealed a strong male-biased sex ratio, suggesting a suboptimal reproductive trend for the *M. sculpturalis* local population. Additionally, we documented for the first time the presence of three natural enemies on the brood of the exotic bee that are common on native co-nesting bees. These novel findings broaden the knowledge on solitary bee invasions.

**Abstract:**

*Megachile sculpturalis* (Smith, 1853) is the first exotic bee species in Europe. Its remarkably fast expansion across this continent is leading to a growing concern on the extent of negative impacts to the native fauna. To evaluate the interactions of exotic bees with local wild bees, we set up trap nests for above-ground nesting bees on a semi-urban area of north-western Italy. We aimed to investigate the interaction in artificial traps between the exotic and native wild bees and to assess offspring traits accounting for exotic bee fitness: progeny sex ratio and incidence of natural enemies. We found that the tunnels occupied by exotic bees were already cohabited by *O. cornuta*, and thus the cells of later nesting alien bees may block the native bee emergence for the next year. The progeny sex ratio of *M. sculpturalis* was strongly unbalanced toward males, indicating a temporary adverse population trend in the local invaded area. In addition, we documented the presence of three native natural enemies affecting the brood of the exotic bee. Our results bring out new insights on how the *M. sculpturalis* indirectly competes with native species and on its performance in new locations.

## 1. Introduction

Introduced non-native bees (*Hymenoptera apoidea*) can enhance pollination service, but they can also have detrimental effects on local ecosystems [1]. Competition for floral resources or nesting sites, diseases transmission and changes in the pollination network are the mechanisms that have been deemed responsible for impacts directly on native bees [2] and indirectly on plant communities [3].

*Megachile sculpturalis* Smith, 1853, native from eastern Asia (China, Korea, Japan), is the first unintentionally introduced bee species in Europe, and it showed a remarkably fast spread across this continent. Since 2008, it expanded from southern France [4] towards eastern Europe [5,6,7,8,9,10], reaching the Crimea peninsula in 2018 [11] and westwards eastern Spain [12,13]. Moreover, according to an evaluation of suitable climatic areas, *M. sculpturalis* is predicted to keep on spreading in most of Europe [14]. Similarly, this species had rapidly colonized the entire eastern half of the USA since its arrival in 1994 [15,16]. 

The fast spread of the *M. sculpturalis* has been linked to some characteristics: the likely wide flight range according to its large body size (18–39 mm in length) [17,18], the passive human-mediated dispersion via traded goods [19], and the wide diet spectrum that includes different flowering plants, such as nectar and pollen sources (polylectic diet) [5,8,20]. In parallel to these aspects, *M. sculpturalis* is well-adapted to colonize anthropogenic environments, since it has a strong preference for ornamental plant pollen [9,12], and it has an opportunistic nesting behavior, as it uses a diversity of pre-existing above-ground cavities regardless of their natural or human origin [11]. Despite the great expansion of the *M. sculpturalis*, the species has also displayed a male-biased sex ratio [21]. This trait is usually associated with a poor reproductive potential [22], and it could be a response to disadvantageous conditions due to eroded genetic diversity, resources shortage, inadequate climatic conditions or parasite pressure [23,24,25,26,27]. Therefore, whether the unbalanced sex ratio is a low fitness response or a generalized trait is still to be unveiled. 

*M. sculpturalis* is a competitor for nesting resources against some native Apoidea species. In fact, the exotic bee has been found to evict pre-existing nesting sites of *Osmia* and *Xylocopa* [10,17,28,29]. Such an antagonistic nesting trait is likely to affect other above-ground nesting species that need a similar nesting substrate (holes in logs, stems, reeds, wooden trap nests) and similar cavity diameter (8–12 mm) [5,21]. A previous study has pointed out that the eviction mechanism may be among the reasons for the negative correlation seen between native bees and the exotic bee in an urban area in south France [21]. Nevertheless, giant resin bee nesting biology has not yet been widely studied, and besides the eviction mechanism, other direct or indirect interactions may be involved in the competition for nesting resources. The competition mechanisms with native bees are particularly important aspects for assessing the hypothesis that *M. sculpturalis* could harm native wild bees in Europe, and novel information on its behavior are essential to have a better understanding of its potential negative impacts.

The host-parasite system is among the factors facilitating the success of an invasion [30]. Parasites might mediate the invasion of an introduced species by modifying native host-parasites relationships [31]. To our knowledge, parasites and natural enemies of *M. sculpturalis* in either its home range or new locations have never been studied.

This study provides new insights on traits linked to *M. sculpturalis* fitness in the new colonized area in Italy where it has been present since 2009 [5], and it adds novel evidence of likely negative effects of this exotic bee on native bee species. Specifically, our aims were: (1) to evaluate the interaction in artificial trap nests, located in Italian study areas, between native bees and the alien bee, (2) to explore the offspring progeny weight and sex ratio as parameters indicating species fitness, also in relation to the sex ratio detected for *M. sculpturalis* populations in the native and colonized areas, and (3) to provide the first report on natural enemies affecting the *M. sculpturalis* brood. 

## 2. Materials and Methods

### 2.1. Study Organism

**Figure 1:**
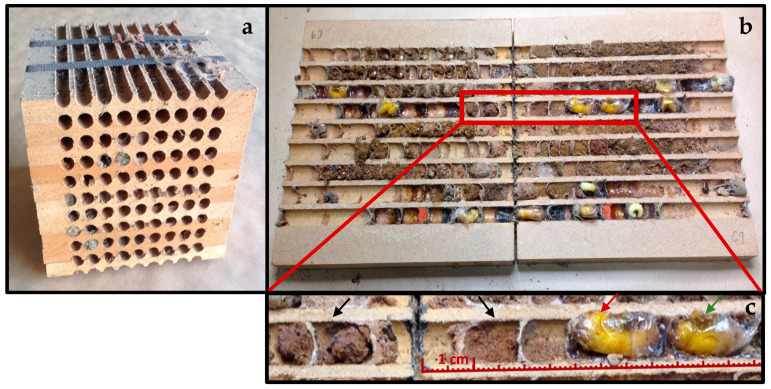
(**a**) Trap nests made of grooved boards stacked together in a solid block. (**b**) Opened individual grooved board showing the upper part (**left**) and lower part (**right**) of the same tunnels. (**c**) Detail of cohabitated tunnel with parasitized *Osmia* cocoons in the inner cells (black arrows), *M. sculpturalis* female prepupa situated next to *O. cornuta* cells (red arrow) and *M. sculpturalis* male prepupa (green arrow).

*Megachile sculpturalis* is a univoltine and protandric species, whose males emerge earlier than females [10]. According to several reports, its nesting season starts in late June to early July and generally ends in mid-September [5,16]. Like most Megachilidae species, it exhibits a sexual size dimorphism in which females are larger than males. This difference is also evident enough in brood cell sizes to allow preliminary gender recognition (Figure 1c). *M. sculpturalis*, like most solitary bees, has a high control over the size and sex of its offspring [22]. It is a precavity nester bee, unable to excavate its own cavities and thus depending on the availability of suitable nesting places [23]. Brood cells and nest closures are created using a mixture of wood fibers, leaf fragments, clay and resin [32] (Figure 1a–c). 

### 2.2. Study Area and Sampling

In January 2018, we placed four trap nests in two towns south of Turin (north-western Italy). The locations were chosen since the presence of *M. sculpuralis* had been reported nearby in previous years. The climate is typically continental, with cold winters and moderate summers [33]. The mean annual temperature is 12.5 °C, and the mean annual rainfall is 900 mm [34]. 

We used two private gardens, one close to the hilly Monte San Giorgio natural park and the other encircled by farmland (municipalities of Piossasco and Volvera, respectively) (Figure S1).

Each trap nest consisted of a medium density fibreboard block of 20 × 20 × 15 cm made by a series of individual boards grooved with channels (also called grooved boards or laminates), which were stacked together to form 81 tunnels of 1 cm diameter (Figure 1). To avoid the effect of the cavity size on the alien bee sex progeny [35,36], all nesting cavities had the *M. sculpturalis* preferred diameter of 9.5–10 mm [5,21], which overlapped with the accepted diameters for other solitary wild bees [36]. Trap nests were secured on walls between 2–4 m above the ground and sheltered from rain. 

Trap nests were opened to analyze their content during late November 2018, when the wild bee nesting season ended. For each nest, we recorded the number of intact brood cells, the number of cells attacked by natural enemies, their position inside the tunnel and the species (if possible). The low diversity of species in trap nests allowed us to identify them during opening. The species’ identity was confirmed after adult emergence. Natural enemies were identified using studies by Fliszkiewicz et al., Krunić et al., Zajdel et al. and Majka et al. [37,38,39,40]. We only kept and reared intact brood cells using specimens that had reached the prepupal or adult stage, depending on the species (Figure 1c). Prepupae and cocoons were then wintered separately in glass vials, in complete darkness, inside an environmental chamber at ambient temperature.

During spring and summer 2019, we checked specimens every three days, and we identified the sex and weight at emergence. When adults did not emerge, we inferred the sex from the cell size and the sex of bees in the neighbouring cells, according to Seidelmann’s methods for protandric solitary bee species [41].

### 2.3. Data Analysis

#### 2.3.1. Offspring Traits and Parasitism

We compared the progeny weight between sexes using a linear model after having log-transformed the weight to reach normality. The model did not improve when taking into account the nest as a random effect, so it was not included. Then, focusing on *M. sculpturalis* progeny, we calculated the observed sex ratio and the expected one according to Fisher’s sex allocation theory. In particular, the theory predicts that the parental investment must be divided equally between sexes in panmictic populations [24,42]. In this context, for sexually dimorphic species, the progeny sex ratio is expected to be proportionally biased toward the sex with the lower investment. 

The expected frequencies were calculated based on Torchio and Tepedino’s formula [23], where the expected sex ratio is equal to the ratio between the mean female weight and mean male weight. The sex ratio and expected frequencies were calculated individually for each trap nest. The comparison of the observed sex ratio with the expected one was tested for each nest through a paired t-test. Furthermore, we aimed to compare these observed and expected sex ratios with the sex ratio recorded by open access global data. We used *M. sculpturalis* distribution data collected from two sources, either entomology collections or field observations, available in the Global Biodiversity Information Facility (GBIF) [43]. Records that did not include sex identification were removed. Then, data were grouped between observations in the native area (China, Korea and Japan) and those placed in the new colonized area (North America and Europe). Records without a location assignment but dated before 1993 were assigned to the native area since the species has never been reported outside its native range before this year [15]. The sex ratio was calculated separately to verify changes in the sex allocation strategy between the native and colonized areas. The proportions of females and males from the two areas were compared through a chi-square test. We assumed that the effect of bias in the sex detection, due to how the GBIF data were collected, was negligible.

The parasitism rate was computed as the ratio between parasite-infected cells divided by overall bees belonging to the same species. 

All statistical analyses were carried out with the R software (version 3.5.1) using the lme4 package [44].

## 3. Results

### 3.1. Above-ground Nesting Community

229 out of a total of 324 available tunnels were occupied by two species, *M. sculpturalis* and *O. cornuta*, and none of the trap nests were colonized by further wild bee species. Overall, 25% of nests were built by *M. sculpturalis*, while 75% were built by *O. cornuta* (Table 1). For both species, the nests consisted of a series of female brood cells (the later-emerging sex) in the inner part of the nest tunnel and a series of males (the earlier-emerging sex) at the entrance. 

### 3.2. Interaction between the Exotic and the Native Bee

We recorded the coexistence of both species cohabitating in 26 tunnels. 44% of tunnels occupied by the exotic bee were built under cohabitation despite empty tunnels being available (Figure S2 and Database in Supplementary Materials).

**Figure 2:**
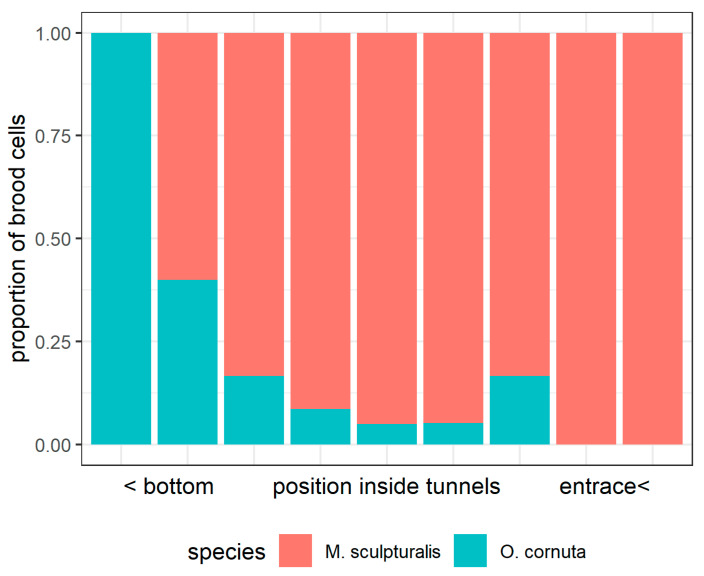
Proportion of species brood cells according to their position inside cohabitated tunnels, with a maximum of nine occupied positions (from the bottom to the entrance).

We observed a maximum number of nine brood cells within the cohabited tunnels. Inner tunnel positions were mostly occupied by *O. cornuta*, while the outer positions (toward the entrance) were filled by *M. sculpturalis* (Figure 1c and Figure 2). 

### 3.3. Progeny Weight and Sex Ratio of Exotic Bee

**Figure 3:**
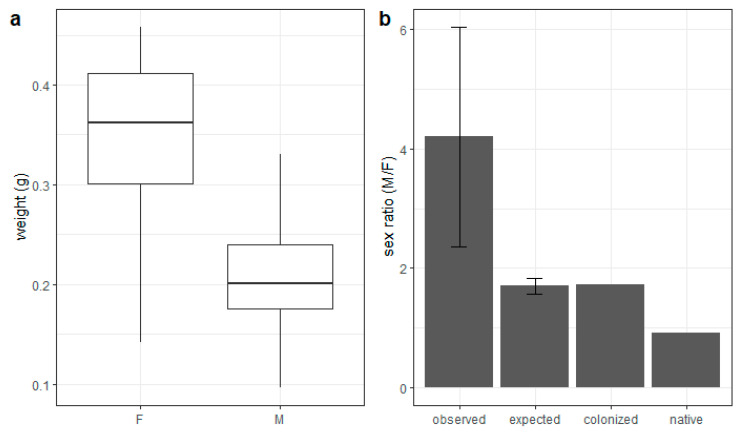
Progeny weight and sex ratio of *M. sculpturalis.* (**a**) Boxplot of female and male weights; (**b**) sex ratios (male/female) and standard error for observed nests, expected sex ratio according to Fischer’s sex allocation theory (see Section 2.3.1. for details) and sex ratio calculated using GBIF data, respectively in the colonized and native areas.

Female *M. sculpturalis* were significantly heavier than males (x male weight = 0.203 g, x female weight = 0.350 g, F = 129.1, df = 201, *p* < 0.001) (Figure 3a). We observed a sex ratio strongly biased toward males, resulting in 4.2 males for each female (Figure 3b and Table A1 in Appendix A). This result was significantly higher than the expected sex ratio estimated by the ratio between male and female weights (t = 3.48, df = 3, *p* = 0.04). Regarding the GBIF database, we found 331 records of *M. sculpturalis* from the native area and 351 from the new colonized area having a sex identification. The sex ratio was 1.72 and 0.91, respectively, in the colonized and native ranges. The former was very close to the predicted Fisher’s sex ratio, while the latter was significantly lower than the sex ratio in the colonized range (χ^2^ = 15.994, df = 1, *p* < 0.001) and, in turn, highly different from the observed sex ratio (Figure 3b).

### 3.4. Natural Enemies

Most *O. cornuta* cells were parasitized, and as a consequence only 12 bees emerged from stored cocoons. In contrast, 213 *M. sculpturalis* adults emerged from 244 brood cells.

Five species of natural enemies were found infesting 93% of *O. cornuta* cells (Table 1). 

**Figure 4:**
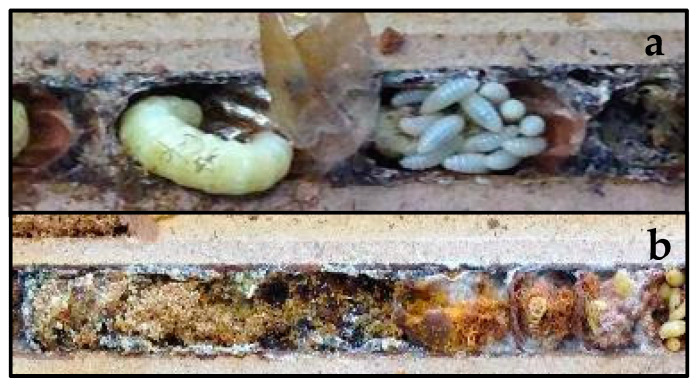
Natural enemies found in *M. sculpturalis* brood cells. (**a**) Prepupae of *Monodontomerus obscurus* (**right**) next to intact *M. sculpturalis* prepupa (**left**). (**b**) Larvae of *Cacoxenus indagator* (right).

Among these natural enemies, three parasitized *M. sculpturalis* as well: *Cacoxenus indagator*, *Monodontomerus obscurus* and *Ptinus sexpunctatus* (Figure 4). However, these enemies were found exclusively in 7% of overall alien bee brood cells.

## 4. Discussion

The fast spreading of *M. sculpturalis* and its aggressive nesting behavior suggested a likely successful invasive performance [8,10,14]. Novel empirical evidence on the interactions and traits of introduced species can indicate if they are thriving in the new location. Moreover, the understanding of how the alien wild bee impacts the native fauna is a challenging and complex issue [1]; hence, investigations that highlight interaction mechanisms are useful for understanding what impacts should be expected. In this study, we found broods of *M. sculpturalis* and *O. cornuta* cohabitating inside the same tunnels. This evidence, together with the different phenologies of the two species, may implicate an interaction mechanism, which negatively affects the native bee. The fact that the exotic bee occupied outer positions (i.e., toward the entrance) in cohabitated tunnels is the result of its later nesting period, following that of *O. cornuta.* In addition, exotic brood cells are sealed with resin and remain locked until the following summer. Consequently, the spring-emerging *O. cornuta* from the inner positions of the tunnel may get trapped, due to the barrier of resin and *M. sculpturalis* cell contents blocking the *Osmia* emergence. Previous studies have consistently demonstrated that *M. sculpturalis* is capable of evicting pre-adult stages of other bees from their cells [10,28,44,45,46]. Thus, our results indicate the possibility of a potential combining effect of direct (eviction) and indirect competition (emergence blocking) acting at different times on the same nests. In this study, we detected the interaction with one native bee species only, probably because of a low richness of cavity-nesting bees in the study areas and the exclusive cavity diameter used in our trap nests. However, it is expected that other species of the genera *Osmia, Anthidium* and *Xylocopa* might be affected by the abovementioned mechanisms [8,47], although emergence blocking should only occur in earlier-emerging species, particularly in *Osmia* sp. 

In the assessment of the progeny sex ratio, *M. sculpturalis* showed a greater male unbalance than expected, based on Fisher’s theory of parental investment and sex allocation [42]. This result is in agreement with the high proportion of males (83%) found by Geslin et al. in southern France [21]. A recent research on the genetic variability of the giant resin bee provides insights into the skewed male sex ratio, as it detected a high percentage of diploid males among individuals sampled in Vienna (Austria) [48]. Diploid males are probably the consequence of a founder effect in new colonized areas. Furthermore, it has been discovered that a low genetic diversity and the associated skewed sex ratio, even if temporarily limiting the performance of invasive species, do not always limit their spread over time, as theoretically expected [49,50]. In particular, in invading social species, the haplo-diploid system is capable of overcoming the issue through multiple introductions [48,51] and natural selection mechanisms, which increases average heterozygosis at the sex locus over time [49]. 

Despite the expectation of a higher male-biased sex ratio as a common pattern in a new colonized area, we found that the sex ratio of the *M. sculpturalis* from global data met the theoretical Fisher’s prediction. Therefore, it seems that the overall exotic population did not suffer from a skewed male sex ratio like our local Italian population and the French one showed. This result also suggests that the unbalanced sex ratio might be a location-dependent limiting factor for the alien bee [52]. Additionally, the sex ratio from native ranges were lower than expected. We believe that our results should be considered as baseline data to verify whether the skewed sex ratio is a factor involved in the *M. sculpturalis* invasion dynamic. 

It has been argued that the success of a biological invasion might be facilitated by the invader species escaping from their natural enemies and by the modification of parasitism relationships in new locations [30]. While a parasite introduction due to the spread of an alien species may occur, invasive species can act as new hosts and also acquire parasites from native species [53]. We detected three generalist natural enemies [53] in the exotic brood cells that were also present in *O. cornuta* cells. Two of them (*Cacoxenus indagator* and *Monodontomerus obscurus)* have a European native range [54], while the third (*Ptinus sexpunctatus*) has a Paleartic distribution, and thus it should be present in the *M. sculpturalis* original range. Our observations provide the first record of parasitism in *M. sculpturalis* in the European territory. Despite potential adverse consequences of this parasite acquisition for the exotic bee, it was the most successful species in terms of emerging adults, and the overall parasitism rate was very low (7%) compared to that of *O. cornuta* cells (93%). This suggested that *M. sculpturalis* was potentially less susceptible to natural enemies than the native bee.

The novelties introduced in this study are essential knowledge on the competitive dynamic between native and alien bees, on species-fitness traits and on the incidence of natural enemies. Our results indicate that the giant resin bee might be a competitor with the native *O. cornuta* for nesting resources. Future studies using nesting traps and long-monitoring data will help to characterize the impacts of this fast-spreading exotic bee.

## Figures and Tables

**Table 1:** Cavity-nesting bees and their natural enemies in trap nests.

Species	Nests (Occupied Tunnels)	Total Brood Cells	Mean Number of Cells per Nest	Maximum Number of Cells per Nest *	% of Parasitized Brood Cells	Emerged Adults	Natural Enemies
*Osmia* *cornuta*	171	1013	5.9	15	93	12	*Cacoxenus indagator* *Chaetodactylus osmiae* *Melittobia acasta* *Monodontomerus obscurus* *Ptinus sexpunctatus*
*Megachile sculpturalis*	58	244	4.9	7	7	213	*Cacoxenus indagator* *Monodontomerus obscurus* *Ptinus sexpunctatus*

* Number of cells within a tunnel belonging to one species only.

## Data Availability

All relevant data is provided in the Supplementary Materials.

## Supplementary Materials

The following will be available online at https://www.mdpi.com/article/10.3390/insects12060545/s1. Figure S1: Map of sampling sites; Figure S2: Graphical nest visualization of species occupation for each tunnel in trap nests; Database: raw data of trap nests is also available.

## Appendix A

**Table A1:** Progeny weight differences and sex ratios in adult *M. sculpturalis*.

Nest	F	Mean Weight (g)	M	Mean Weight (g)	Observed Sex Ratio	Expected Sex Ratio
A	11	0.353	46	0.205	4.2	1.7
B	10	0.352	39	0.215	3.9	1.6
C	15	0.341	53	0.199	3.5	1.7
D	4	0.370	30	0.199	7.5	1.9
Total	40	0.350	168	0.203	4.2	1.7
