# Supplementary material for: Nesting, Sex Ratio and Natural Enemies of the Giant Resin Bee in Relation to Native Species in Europe
Source: Insects. 2021 Jun 11;12(6):545. doi: 10.3390/insects12060545 (PMC8230627; doi:10.3390/insects12060545)
Supplement: Supplementary file 1 [file insects-12-00545-s001.zip › Database_Trap_nests.pdf]

NEST	FLOOR	TUNNEL	OSMIA CELLS	OSMIA PARASITIZED	MEGACHILE CELLS	MEGACHILE PARASITIZED
A	1	1	0	0	0	0
A	1	2	0	0	0	0
A	1	3	0	0	0	0
A	1	4	0	0	0	0
A	1	5	0	0	0	0
A	1	6	0	0	0	0
A	1	7	0	0	0	0
A	1	8	0	0	0	0
A	1	9	0	0	0	0
A	2	1	5	3	0	0
A	2	2	4	4	0	0
A	2	3	11	8	0	0
A	2	4	0	0	3	0
A	2	5	0	0	1	0
A	2	6	0	0	0	0
A	2	7	0	0	0	0
A	2	8	0	0	0	0
A	2	9	2	1	0	0
A	3	1	10	9	0	0
A	3	2	8	8	0	0
A	3	3	4	4	0	0
A	3	4	0	0	6	0
A	3	5	3	3	3	0
A	3	6	0	0	0	0
A	3	7	0	0	0	0
A	3	8	1	1	0	0
A	3	9	10	0	0	0
A	4	1	0	0	0	0
A	4	2	6	6	0	0
A	4	3	2	2	0	0
A	4	4	2	2	0	0
A	4	5	4	4	0	0
A	4	6	0	0	0	0
A	4	7	0	0	0	0
A	4	8	9	9	0	0
A	4	9	0	0	0	0
A	5	1	0	0	3	0
A	5	2	6	6	0	0
A	5	3	6	6	0	0
A	5	4	1	0	0	0
A	5	5	2	2	0	0
A	5	6	7	3	0	0

A	5	7	0	0	0	0
A	5	8	0	0	0	0
A	5	9	2	2	0	0
A	6	1	1	1	5	0
A	6	2	0	0	6	0
A	6	3	0	0	0	0
A	6	4	1	1	0	0
A	6	5	4	4	0	0
A	6	6	8	8	0	0
A	6	7	8	1	0	0
A	6	8	5	2	0	0
A	6	9	2	2	0	0
A	7	1	0	0	5	0
A	7	2	0	0	2	0
A	7	3	0	0	1	0
A	7	4	0	0	5	0
A	7	5	0	0	4	0
A	7	6	0	0	0	0
A	7	7	0	0	0	0
A	7	8	1	1	0	0
A	7	9	4	4	0	0
A	8	1	9	9	0	0
A	8	2	0	0	6	0
A	8	3	0	0	5	0
A	8	4	0	0	5	0
A	8	5	0	0	0	0
A	8	6	0	0	0	0
A	8	7	9	9	0	0
A	8	8	4	4	0	0
A	8	9	1	1	0	0
A	9	1	0	0	5	3
A	9	2	0	0	0	0
A	9	3	0	0	0	0
A	9	4	0	0	0	0
A	9	5	0	0	0	0
A	9	6	0	0	0	0
A	9	7	8	8	0	0
A	9	8	3	3	0	0
A	9	9	0	0	0	0
B	3	1	6	6	0	0
B	3	2	12	12	0	0
B	3	3	10	10	0	0
B	3	4	2	2	0	0

B	3	5	10	10	0	0
B	3	6	10	10	0	0
B	3	7	3	3	0	0
B	3	8	6	5	0	0
B	3	9	13	13	0	0
B	1	1	8	8	0	0
B	1	2	15	15	0	0
B	1	3	0	0	0	0
B	1	4	0	0	0	0
B	1	5	0	0	0	0
B	1	6	0	0	0	0
B	1	7	0	0	0	0
B	1	8	0	0	0	0
B	1	9	0	0	0	0
B	2	1	5	5	0	0
B	2	2	9	9	0	0
B	2	3	1	0	3	0
B	2	4	1	0	5	0
B	2	5	2	1	0	0
B	2	6	5	5	0	0
B	2	7	9	9	0	0
B	2	8	9	9	0	0
B	2	9	9	9	0	0
B	4	1	12	12	0	0
B	4	2	2	2	0	0
B	4	3	3	3	5	2
B	4	4	1	1	5	0
B	4	5	3	3	0	0
B	4	6	4	2	0	0
B	4	7	10	9	0	0
B	4	8	11	11	0	0
B	4	9	9	9	0	0
B	5	1	7	7	0	0
B	5	2	2	2	5	0
B	5	3	6	6	0	0
B	5	4	3	3	0	0
B	5	5	1	1	1	0
B	5	6	1	1	3	0
B	5	7	1	1	3	0
B	5	8	10	10	0	0
B	5	9	11	11	0	0
B	6	1	9	9	0	0
B	6	2	1	1	5	0

B	6	3	2	2	0	0
B	6	4	3	3	0	0
B	6	5	1	1	0	0
B	6	6	1	1	0	0
B	6	7	0	0	5	0
B	6	8	9	9	0	0
B	6	9	7	7	0	0
B	7	1	2	2	0	0
B	7	2	1	1	0	0
B	7	3	3	3	0	0
B	7	4	9	9	0	0
B	7	5	5	4	0	0
B	7	6	1	1	5	0
B	7	7	0	0	4	0
B	7	8	3	3	0	0
B	7	9	1	1	0	0
B	8	1	9	9	0	0
B	8	2	2	1	0	0
B	8	3	11	11	0	0
B	8	4	3	3	0	0
B	8	5	2	2	0	0
B	8	6	1	1	5	0
B	8	7	11	10	0	0
B	8	8	11	10	0	0
B	8	9	13	13	0	0
B	9	1	12	11	0	0
B	9	2	0	0	0	0
B	9	3	1	1	0	0
B	9	4	3	3	0	0
B	9	5	0	0	0	0
B	9	6	8	8	0	0
B	9	7	4	4	0	0
B	9	8	4	4	0	0
B	9	9	10	3	0	0
C	4	1	6	6	0	0
C	4	2	0	0	5	5
C	4	3	10	10	0	0
C	4	4	10	10	0	0
C	4	5	7	5	0	0
C	4	6	8	8	0	0
C	4	7	12	12	0	0
C	4	8	5	5	0	0
C	4	9	15	15	0	0

C	1	1	8	8	0	0
C	1	2	0	0	0	0
C	1	3	0	0	0	0
C	1	4	3	3	0	0
C	1	5	4	4	0	0
C	1	6	2	2	0	0
C	1	7	2	2	0	0
C	1	8	10	10	0	0
C	1	9	12	12	0	0
C	2	1	0	0	0	0
C	2	2	0	0	0	0
C	2	3	0	0	0	0
C	2	4	0	0	0	0
C	2	5	0	0	0	0
C	2	6	0	0	0	0
C	2	7	0	0	0	0
C	2	8	0	0	3	0
C	2	9	6	6	0	0
C	3	1	13	13	0	0
C	3	2	4	4	4	0
C	3	3	11	10	0	0
C	3	4	11	11	0	0
C	3	5	12	12	0	0
C	3	6	8	8	0	0
C	3	7	1	1	0	0
C	3	8	1	1	0	0
C	3	9	10	10	0	0
C	5	1	9	9	0	0
C	5	2	1	1	5	4
C	5	3	3	3	5	1
C	5	4	1	1	5	0
C	5	5	7	5	0	0
C	5	6	3	3	0	0
C	5	7	0	0	0	0
C	5	8	8	8	0	0
C	5	9	7	7	0	0
C	6	1	9	8	0	0
C	6	2	1	1	6	1
C	6	3	0	0	6	1
C	6	4	4	4	0	0
C	6	5	2	2	0	0
C	6	6	0	0	5	0
C	6	7	7	7	2	2

C	6	8	1	0	0	0
C	6	9	2	2	4	0
C	7	1	12	10	0	0
C	7	2	0	0	5	0
C	7	3	0	0	5	0
C	7	4	3	3	0	0
C	7	5	1	0	3	0
C	7	6	13	12	0	0
C	7	7	12	11	0	0
C	7	8	11	10	0	0
C	7	9	6	4	0	0
C	8	1	9	8	0	0
C	8	2	0	0	6	0
C	8	3	0	0	5	0
C	8	4	11	11	0	0
C	8	5	1	1	6	0
C	8	6	4	3	0	0
C	8	7	10	10	0	0
C	8	8	10	10	0	0
C	8	9	3	3	0	0
C	9	1	0	0	5	0
C	9	2	2	2	1	0
C	9	3	12	12	0	0
C	9	4	10	9	0	0
C	9	5	7	7	0	0
C	9	6	2	2	4	0
C	9	7	7	7	2	0
C	9	8	14	14	0	0
C	9	9	9	9	0	0
E	1	1	0	0	0	0
E	1	2	0	0	0	0
E	1	3	0	0	0	0
E	1	4	0	0	0	0
E	1	5	0	0	0	0
E	1	6	0	0	0	0
E	1	7	0	0	0	0
E	1	8	0	0	0	0
E	1	9	0	0	0	0
E	2	1	0	0	0	0
E	2	2	0	0	0	0
E	2	3	0	0	0	0
E	2	4	0	0	0	0
E	2	5	0	0	0	0

E	2	6	0	0	0	0
E	2	7	0	0	0	0
E	2	8	0	0	0	0
E	2	9	0	0	0	0
E	3	1	0	0	0	0
E	3	2	0	0	0	0
E	3	3	0	0	0	0
E	3	4	0	0	0	0
E	3	5	0	0	0	0
E	3	6	0	0	0	0
E	3	7	0	0	0	0
E	3	8	0	0	0	0
E	3	9	0	0	0	0
E	4	1	0	0	0	0
E	4	2	0	0	0	0
E	4	3	0	0	0	0
E	4	4	0	0	0	0
E	4	5	0	0	0	0
E	4	6	0	0	0	0
E	4	7	0	0	0	0
E	4	8	0	0	0	0
E	4	9	0	0	0	0
E	5	1	0	0	0	0
E	5	2	0	0	0	0
E	5	3	0	0	0	0
E	5	4	0	0	0	0
E	5	5	0	0	0	0
E	5	6	0	0	0	0
E	5	7	0	0	0	0
E	5	8	0	0	0	0
E	5	9	0	0	0	0
E	6	1	0	0	0	0
E	6	2	0	0	0	0
E	6	3	0	0	0	0
E	6	4	0	0	0	0
E	6	5	0	0	0	0
E	6	6	0	0	0	0
E	6	7	0	0	0	0
E	6	8	0	0	0	0
E	6	9	0	0	0	0
E	7	2	0	0	0	0
E	7	3	0	0	0	0
E	7	4	0	0	0	0

E	7	6	0	0	0	0
E	7	7	0	0	0	0
E	7	8	0	0	0	0
E	7	9	0	0	0	0
E	8	3	0	0	0	0
E	8	5	0	0	0	0
E	8	6	0	0	0	0
E	8	7	0	0	0	0
E	8	9	0	0	0	0
E	9	1	0	0	0	0
E	9	2	0	0	0	0
E	9	3	0	0	0	0
E	9	8	0	0	0	0
E	7	1	7	6	0	0
E	7	5	1	1	0	0
E	8	1	2	2	4	0
E	8	2	0	0	6	0
E	8	4	0	0	1	0
E	8	8	0	0	0	0
E	9	4	0	0	3	0
E	9	5	0	0	2	0
E	9	6	0	0	4	0
E	9	7	0	0	6	0
E	9	9	0	0	7	0
