# Supplementary material for: Nesting, Sex Ratio and Natural Enemies of the Giant Resin Bee in Relation to Native Species in Europe
Source: Insects. 2021 Jun 11;12(6):545. doi: 10.3390/insects12060545 (PMC8230627; doi:10.3390/insects12060545)
Supplement: Supplementary file 1 [file insects-12-00545-s001.zip › Supplementary material_with_map.pdf]

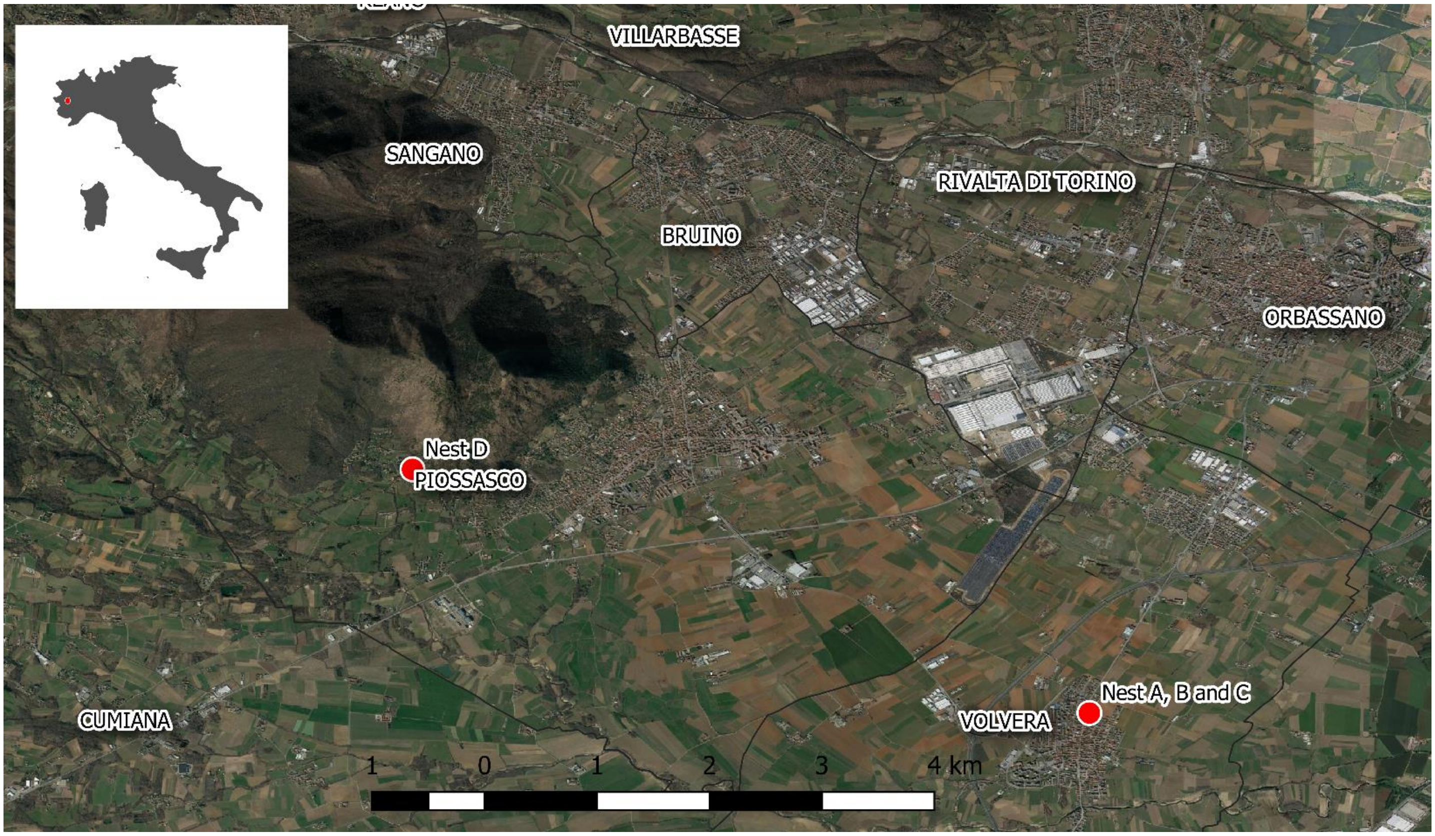

Supplementary Figure S1. Location of sampling sites (red dots) in Turin Province, Italy. The area is characterized by highly intensive crops in lowlands consisting mainly of cereals (wheat, barley and corn) and pome fruits (Malus domestica, Pyrus communis). Fragments of natural and semi-natural habitats are also present in low proportions in hills and mountains. In these, the main vegetation type is the oak forest. In both inhabited and cultivated areas, plants that provide abundant nectar and pollen are *Prunus spp*., *Robinia pseudoacacia*, *Medicago sativa*, *Rubus spp*. and a variety of other ornamental plants such as *Ligustrum spp*., *Hybiscus sp*. and *Cytrus spp.*

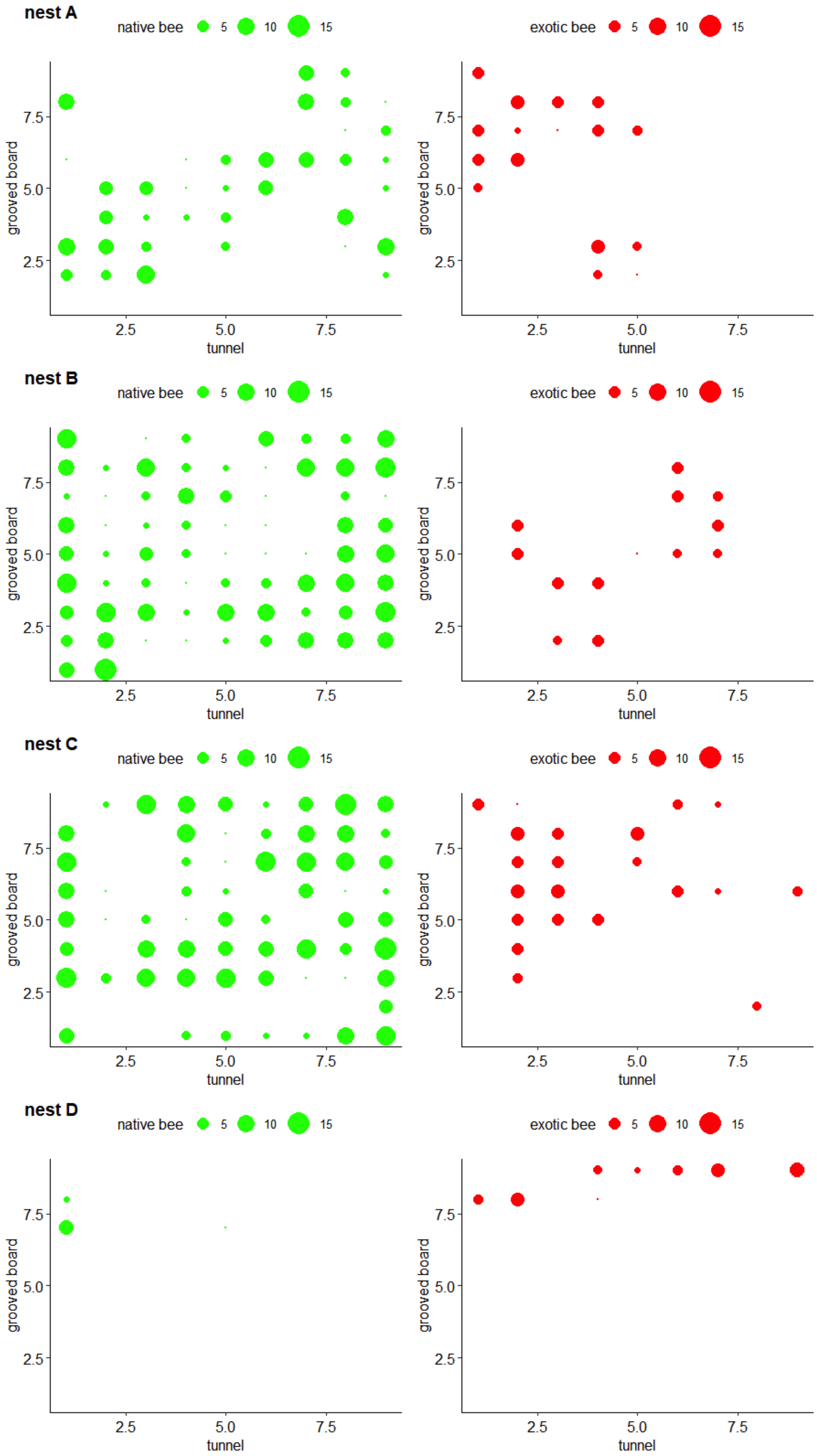

Supplementary Figure S2. Graphical nest visualization of species occupation for each tunnel within four trap nests (A, B, C, D). Green dots represents native bee (*O.cornuta*) occupation, while red dots represents exotic bee (*M. sculpturalis)* occupation. Dimension of dots indicate abundance.
